# Laboratory and In-Situ Testing of Integrated FBG Sensors for SHM for Concrete and Timber Structures

**DOI:** 10.3390/s20061661

**Published:** 2020-03-17

**Authors:** Kristýna Čápová, Lukáš Velebil, Jan Včelák

**Affiliations:** University Centre for Energy Efficient Buildings, CTU in Prague, Buštěhrad 273 43, Czech Republic; lukas.velebil@cvut.cz (L.V.); jan.vcelak@cvut.cz (J.V.)

**Keywords:** mechanical strain monitoring, SHM, FBG sensors, concrete, bridge structure, testing

## Abstract

Long-term structural health monitoring (SHM) plays an important role in the safety of public transport infrastructure such as bridges or tunnels and warns in the event of any emerging problem. This article describes development and testing of system based on fiber Bragg grating (FBG) sensors that can detect changes in strain and temperature. The first phase of the research has been focused on the development of new fiber optic sensors for the monitoring of concrete structures and their investigation in laboratory conditions. The work also shows novel applicability of the same FBG technology for glulam structures. Mechanical loading tests of the concrete beam as well as glulam beam with embedded sensors were carried out. Data measured by developed fiber optic sensors were compared with the readings from reference sensors as well as with the analytically calculated values. The achieved results proved good agreement between the measured data, analytical data and reference methods. In second phase of the research, the pilot installation of the sensors was carried out on the newly constructed prestressed-concrete bridge. The bridge was monitored throughout pre-stressing phase and monitoring continued after the completion of the construction works. Problems with the fragility of the sensors occurred during the measurements, but the obtained results provide a good basis for further improvement of the system.

## 1. Introduction

As far as public safety is concerned, the long-term structural health monitoring (SHM) of infrastructure, such as bridges or tunnels or, considering public buildings, roof structures, plays a crucial role. Well provided SHM is able to detect the progress of more or less serious damage and warn of extreme negative situations (oncoming collapse of the structure). Nowadays, the designer of load-bearing structure has several options of the SHM system design. This article describes an embedded system based on fiber Bragg grating (FBG) sensors, which is able to detect changes in strain and temperature. FBG sensor is based on a periodic variation in the refractive index of the fiber core (Bragg grating), which reflects particular wavelengths of light in a form of narrow Gaussian peaks, while the rest is transmitted. The Bragg grating frequency and refractive index are sensitive to changes in strain and temperature. The principles of FBG sensing are described in more detail, for example, in [1,2,3].

The fiber Bragg grating technique is usable for a wide spectrum of applications and applicable to different investigated materials. The application of the FBG sensors on concrete [4,5,6] or steel structures [7] is not new, but still not used very often. Although there are some works related to application of fiber optic sensors (FOS) in timber structures such as glue-laminated timber (glulam) [8], it is still very innovative new approach and its industrially applicable process is presented in this work. The bare optical fibers with FBG are very fragile and susceptible to damage. A possible solution is therefore to place the fiber on a suitable plate or to encapsulate it in a special sleeve. Hideaki et al. [7] examined a series of FBG-based sensors for measurement of strain, temperature and displacement and used a sensor multiplexing capability for monitoring of multi-storey steel frame building. Strain sensor was placed on base metal made of stainless steel, temperature sensor was glued to an aluminum plate and displacement sensor was glued to a metal bar. Li et al. [4] applied steel-tube packed FBG sensors to structural monitoring of tall concrete building since the date of its construction with temperature history monitoring within concrete including pouring and curing process. For better strain transfer from concrete to FBG, it is also possible to modify the shape of the sensor and add anchoring flanges to its ends [5]. Optical fibers with FBG can also be embedded in a 3D printed structure, enabling the creation of a suitable shape for a variety of applications [6].

FBG sensors are increasingly used for monitoring of historical and new wooden structures. In the case of historical structures monitoring, the sensors can be attached to the surface of the element as described by Li et al. [9], inserted together with a carbon fiber-reinforced polymer (CFRP) laminae into the cut-out in the wood element, [10], or embedded into a glued joint between lamellas of glued laminated timber, [11]. Installation techniques for FBG sensors with and without physical attachment to the wood laminates using structural and non-structural packages have been developed by Wacker et al. [8] who proved correct operation of its system in laboratory conditions. The industrial application of the FBG system in a timber structure is an innovative step which shifts the laboratory use of the FBG system to industrially applicable technologies for civil engineering. The applicability in standardized processes during construction is verified and shown in this paper for the case of a timber structure GLT and reinforced concrete structure.

Concerning the health monitoring of concrete structures, there are several cases of application of fiber Bragg grating sensors on the surface of the load bearing structure, e.g., existing bridge, particularly in bad technical condition [12,13,14,15]. Gebremichael et al. [16] tested a multiplexed FBG sensor system enabling monitoring of dynamic load events on a 346-m road bridge in Norway. As vertical displacement is a very important parameter defining the load bearing capacity of a bridge deck, Bonopera et al. [17] proposed a set of FBG differential settlement measurement sensors connected by a hydrostatic leveling system of communicating vessels and implemented the system on bridge structure in Taiwan. The pedestrian bridge at the Princeton University Campus has been instrumented by long-gauge FBG strain sensors and by distributed Brillouin Optical Time Domain Analysis (BOTDA) strain sensors during the construction process in order to provide structural health monitoring of the structure, [18]. FBG sensors can be used not only for building construction monitoring but also for real-time monitoring of road and railway traffic [19,20,21,22].

Due to the recent increase in the demand of structural health monitoring of load-bearing elements of various structures, there is a growing need of a simple, robust, environmentally resistant and easy to apply sensor system solution. The integration of the FBG sensor directly into the structure increases the level of protection and assures long-term reliability of the system. This article presents such a system of fiber optic sensors to be embedded directly to the load-bearing structure elements from concrete or timber during their production. The system is introduced by laboratory load and stability tests of a simple reinforced concrete beam, and the description of on-site deployment in the prestressed concrete bridge structure follows [23]. The industrial applicability of the fiber optic system for SHM of glued laminated timber beams is presented [24,25].

## 2. Materials and Methods

### 2.1. Laboratory Testing of Reinforced-Concrete Beam

**Figure 1:**
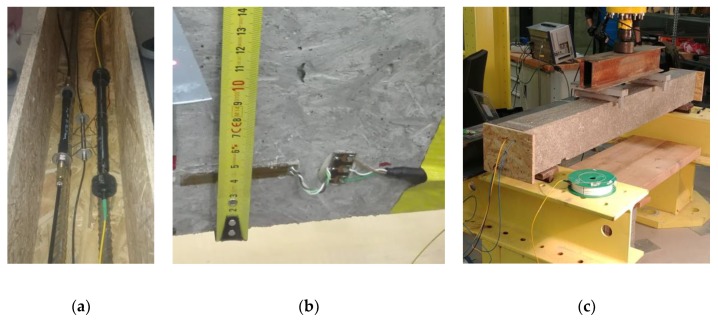
Photos from the installation of the sensors: (**a**) FBG sensors placed in the formwork before concreting the specimen; (**b**) Position of strain gauge on the surface of the specimen; (**c**) The test concrete specimen prepared for the four-point bending test.

The first phase of the research focused on the continued development of new type fiber optic sensors for the monitoring of concrete structures and their investigation in laboratory conditions. Two types of fiber optic sensors were tested: sensor embedded inside a special package and sensor inside a 3D printed structure. The proper sensors function was tested and the obtained data were compared to reference measurements by resistive strain gauge HBM 1-LY41-50/120, commercially available strain sensor HBM FS62, and to the results of calculations by the analytical approach. Three different types of fiber optic sensor were integrated into structure of monolithic reinforced concrete test beam: the tested embedded sensor, the tested 3D printed sensor and the reference HBM strain sensor, see Figure 1a. All the sensors were placed in the lower part of the beam. The tested embedded and 3D printed sensors were attached near the upper surface of steel bar reinforcement. The reference HBM sensor was placed in the middle between the reinforcement bars. All the sensors were attached to the rebars and to the formwork by steel wire in order to secure their position during the process of pouring the fresh concrete into the formwork. Each sensor is equipped with one fiber Bragg grating situated in the middle of the beam span. The sensors are connected with the passive optical cables which have a massive protective secondary coating. The passive cables are used for the signal transmission to the measuring unit. All the optical connectors are of FC/APC type. The sensors are connected to the interrogation unit in series—all the central wavelengths of FBGs had to be chosen very carefully in order not to overlap their measurement ranges. 

**Figure 2:**
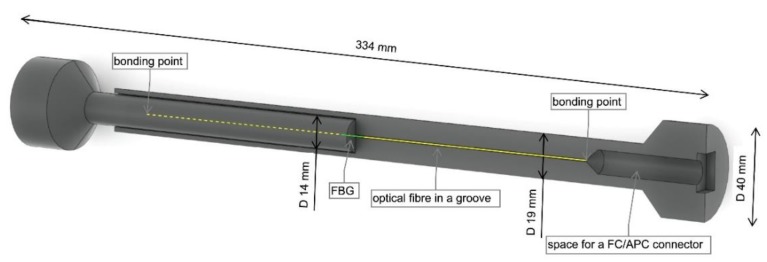
3D model of a 3D printed sensors. One half (on the left) is full, the other half (on the right) is shown in section.

The embedded FBG sensor is designed to withstand the high moisture, temperature and pressure generated during the concreting process and subsequent hardening of the concrete mixture. Optical fiber equipped with the FBG is placed inside a sensor in a special reinforced tube, which also allows prestressing of the sensor for eventual installation in compression zones of concrete elements. The sensor measures the elongation or shortening between two anchor points corresponding to a pair of circular steel plates. The 3D printed sensor is made from two mutually sliding cylinders made of a high strength 3D printing material: thermoplastic ABS-M30. An optical fiber with FBG is located inside the sensor in a groove and mechanically constrained by FC/APC connectors glued to the 3D printed structure. The length of the sensor is 334 mm, the gauge length is 276 mm. The 3D model of the sensor is presented in Figure 2. 

The critical point of the sensing system is where the optical cables leave the concrete specimen – they can be easily broken by a sharp edge of concrete. Therefore, optical cables must be covered by a protective plastic sleeve at this point. During the experiment, reference strain gauge was placed in the midspan on the surface of the beam and glued by adhesive HBM X60, see Figure 1b. The position of the axis of the strain gauge is 45 mm from the bottom surface of the beam. The 50 mm long strain gauge was chosen considering the size of the largest aggregates in the concrete. The reinforced concrete test beam, shown in Figure 1c, with dimensions 150 × 200 × 1800 mm was made of concrete C30/37 and reinforced by two structural steel B500B bars with 20 mm diameter. The tested specimen was produced in December 2017. The compressive strength of concrete was determined by tests of standardized concrete cylinders and cubes after hardening of the concrete mixture.

**Figure 3:**
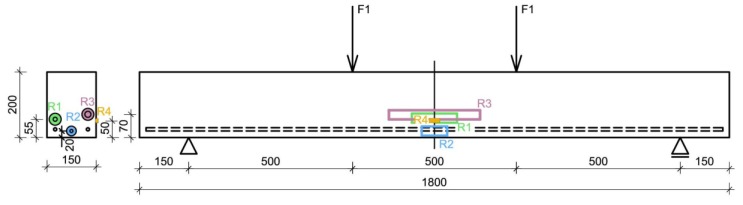
Schematic cross and longitudinal sections of the concrete test beam with the position of sensors, where total force *F* = 2 × *F*1 and specimen span *l* is 1500 mm. R1—tested embedded FBG sensor, R2—reference HBM sensor, R3—tested 3D printed sensor, R4—reference strain gauge.

During the experiment, the specimen was loaded by hydraulic cylinder Inova AHS 630-130 clamped in a steel frame; loading tests were controlled by the cylinder force. A lever designated for the correct distribution of the hydraulic cylinder force to two separate identical forces acting on the concrete specimen was made of steel I profiles and was placed between the cylinder and the specimen. The specimen was simply supported during tests; it was placed on roller bearings. The specimen having a span of 1500 mm was loaded in bending by two forces applied in distances of 500 mm. The arrangement of the test with the position of the sensors is presented in Figure 3.

The concrete beam was subjected to two four-point bending tests. Both tests were carried out consecutively in one day on March 15, 2018. The first test was designed completely in the elastic part of the working diagram and represented cyclic loading with 10 identical cycles. Therefore, the specimen was loaded to 9% of the maximum load-bearing capacity, which was estimated to be 130 kN. Each load cycle consisted of two stages. In the first stage, the beam was loaded at a rate of 0.3 kN/sec to achieve 5.8 kN. After the stabilization of applied force, the sample was completely unloaded (the applied force reached zero value). In the second stage, the beam was loaded at the same rate to achieve 11.6 kN, and then unloaded to zero value. The second test consisted of a cyclic loading with the load gradually increasing until reaching the failure of the specimen. The loading rate was the same as for the first test (0.3 kN/sec). At first, the beam was loaded in three identical cycles. Each cycle had two stages – in the first stage the sample was loaded to 10% and in the second stage to 20% of its maximum load-bearing capacity. In both cases, after force stabilization, the sample was unloaded to zero loading force. After the three two-staged cycles, every following load cycle was designed to always apply about 20% of the load capacity more than in the previous cycle.

The wavelength reflected by the FBG sensors, the resistance of the strain gauge and the force applied by the hydraulic cylinder were recorded during the tests. The values of the applied force were used to determine the strain analytically. For the analytical approach, two different calculation models were used. The first model has been based on the assumption of concrete behavior in the elastic part of its working diagram. Cross section module of beam is given as:

(1) $$W=\frac{bh^{2}}{6}$$

where *b* is the cross-section width and *h* is its height. The bending moment in the middle of the beam span at the four-point bending test is written as:

(2) $$M_{max}=\frac{Fl}{6}$$

where *F* is the total force applied to the test specimen and *l* is the specimen span. Then the maximum stress in the beam cross section is:

(3) $$\sigma_{max}=\frac{M}{W}=\frac{Fl}{bh^{2}}$$

According to Hook’s law:

(4) σ=Eε

where *E* is modulus of elasticity, the strain in the location of the maximum stress can be expressed as:

(5) $$\varepsilon_{max}=\frac{Fl}{bh^{2}E_{cm}}$$

This formula is valid for the maximal value of strain in the cross-section, that means the strain at the bottom fibers of the cross section is calculated (in the maximal distance from the neutral axis). However, none of the tested and reference sensors is placed in the location with the maximal strain; in the elastic part of the working diagram the strain distribution is linearly growing from zero value at the neutral axis to the maximal value at the bottom fibers of the cross section. Therefore, the analytical calculations for the comparison with the values measured by the sensors are linearly interpolated according to the calculation model described in the paragraph above. The second calculation model is valid for loading outside the elastic part of the working diagram; it is a simplified model based on the interpolation between the maximal elastic strain (maximal stress for concrete with no cracks yet) and strain at the point of failure.

### 2.2. Field Test—Prestressed Concrete Bridge

The pilot deployment of the FBG sensors took place in October 2017 due to the seized opportunity of installation the sensors directly into the structure of a newly constructed road bridge which transfers the traffic across the local river in the southern part of the Czech Republic near the village of Staré Hobzí. The load-bearing structure of the bridge is prestressed continuous beam which has three individual spans of 23, 30, and 23 m made of C35/45-XF2 concrete with Y1860-S7-15.7 tendons and B500B structural steel rebars. The load-bearing structure is 77.8 m long and 8.5 m wide with a depth pitching from 1.0 m in the middle to 1.6 m above the supports. 

**Figure 4:**
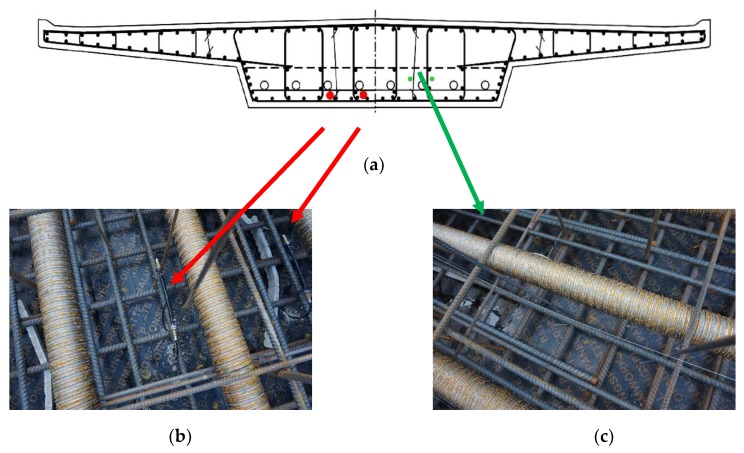
(**a**) Schematic cross section of the load-bearing structure of the bridge. Red dots and arrows: two embedded FBG sensors, (**b**) their position and picture from the installation. Green dots and arrow: GFRP sensor array installed in an open loop, (**c**) its position and picture from the installation.

Two different types of fiber optic sensors were installed into the bridge structure: the embedded FBG sensor described in the previous chapter and the fiber optic sensor array for point measurement of strain in protective glass fiber reinforced polymer (GFRP) sleeve. The outer diameter of the GFRP sensor array is 1 mm, and the longitudinal glass fibers combined with vinyl ester resin protect optical fibers at temperatures in the range from −40 to 120 °C. The installed sensor contained four fiber Bragg gratings. There were two pieces of the embedded FBG sensor attached to the steel reinforcement of the bridge structure and one GFRP sensor array with four measuring points placed into the formwork in an open loop (having U-shape in the layout), see Figure 4. After the installation of the sensors was completed, the bridge was concreted. 

All the measurements and the first level of data processing were provided by the interrogation unit FBGuard 1550 developed by the company Safibra, Ltd. As described in [26,27], FBGuard 1550 is a compact, field proven, industrial grade FBG monitoring unit designed for reliable and long-term operation in 24/7 mode. The FBGuard 1550 unit is completely autonomous with an embedded PC and web server, it contains a broadband light source in the range of 1550 nm and performs spectral analysis of the light by means of a linear array detector and a spectrometer platform. The FBGuard 1550 interrogator has a wavelength resolution ≤1 pm and a wavelength repeatability ±5 pm. The interrogation unit is produced in several variants depending on the number of channels (1, 2, 4, 8 or 16). The maximal scan frequency is up to 11 kHz at 1 channel, 1 kHz at 2 channels, 500 Hz at 4 channels, 250 Hz at 8 channels, and 125 Hz at 16 channels. The durability of optical switch is more than 10^11^ cycles.

### 2.3. Laboratory Testing of Timber Beam

This research focused on the use of fiber optic sensors for the monitoring of concrete structures is based on previous research in which the authors had dealt with the monitoring of wooden structures. For the purposes of laboratory investigations, fiber optic sensors were glued into the structure of glued laminated timber beam.

**Figure 5:**
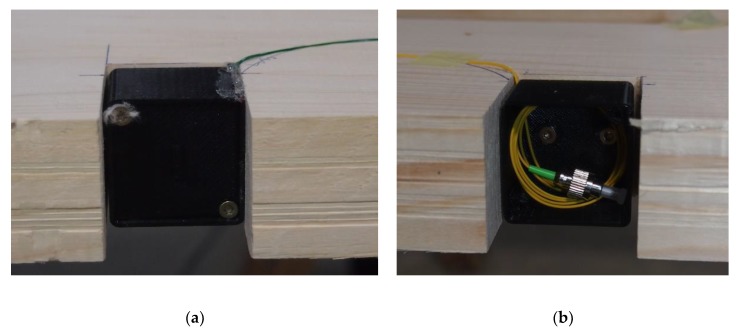
The plastic box inside the cutout of the wooden lamella: (**a**) Box covered by plastic lid; (**b**) Box without the lid with the FC/APC connector inside.

The glued laminated beam of dimensions 140 × 315 × 6110 mm is composed of 8 wooden lamellae. Three optical fibers are placed between the lamellae; each fiber contains 3 FBG sensors working at different wavelengths. The first optical fiber is glued between the two top lamellae, the second is placed between the lamellae at the mid-height of the cross section, and the third is placed between the two bottom lamellae. Optical fibers are embedded in grooves 1 × 2 mm formed in the longitudinal axis of the beam and glued point-wise by a small amount of cyanoacrylate adhesive. The grooves allow easier fixation of the fiber and ensure that it does not move during the lamination process. GLT beams are often planed on all sides during the manufacturing. In order to allow the integration of the fibers to be a part of the standard production process of GLT, the fiber connectors must be hidden inside the GLT. Therefore, cutouts of 40 × 45 mm are created in the respective wooden lamellae in places where the grooves with embedded optical fibers come out to the surface of the GLT beam. The FC/APC connectors are placed inside 3D printed plastic boxes which fit into the prepared cutouts, see Figure 5. Inside the beam, the fibers are well protected by the mass of the wood, but at the point where the fibers come out of the wood surface into the boxes, they are more susceptible to mechanical damage and can be easily broken during handling. This is the reason why the optical fibers are in such places, as well as at points of splice connections, covered by a more massive secondary protection. The boxes with fiber connectors are then covered with plastic lids, see Figure 5a. The connectors are then protected against the gluing effects during the lamination process.

**Figure 6:**
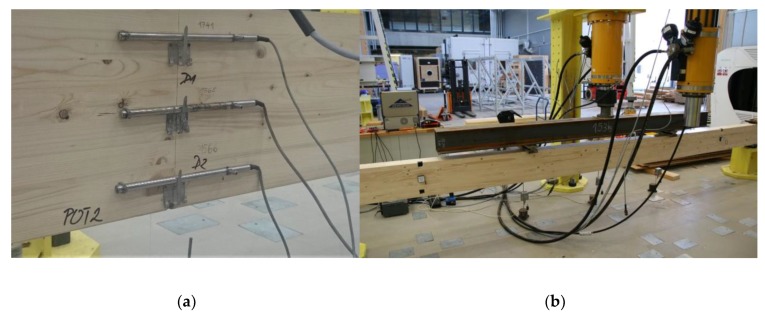
Full size timber specimen: (**a**) Placing the fiber in a wooden lamella during manufacturing process of the beam; (**b**) Test specimen prepared for the four-point bending test.

The data obtained from the optical sensors measurement are verified by reference measurements using extensometers, analytical calculation procedure and in the case of one of the tests also by means of digital image correlation method. Extensometers provide a very accurate measurement of the change in the distance between their measuring points. The initial distance of measuring points during the experiments was 200 mm. Three extensometers HBM D1 were mounted on the side surface of the beam in the middle of the span, see Figure 6a. The positions of the extensometers correspond to the positions of the FBG sensors inside the beam. The analytical calculation method is based on the dependence of the strain on the mechanical stress and the material modulus of elasticity. Therefore, the values of the wood modulus of elasticity in bending were determined from the beam deformation to an applied force dependence in the first load cycle of each test according to EN 408 [28]. Vertical displacements of the beam were measured by potentiometric path sensors during the experiment; the sensors were placed in the middle of the span and under load forces. Digital image correlation was used in order to analyze strain fields on a timber specimen surface. DIC operates on the principle of finding a conformity between small points in a reference image and in images with deformed state. Therefore, a contrasting pattern has been applied by spraying on one side surface of the beam. A Canon 70D high-definition digital camera, fixed on a tripod taking pictures at 3-second time intervals, was used for test. The timber beam equipped with sensors was subjected to cyclic loading by four-point bending test, see Figure 6b. The loading was not carried out to failure of the specimen because of its repeatability and possibility of verification the stability of the optical sensors over time. The test was repeatedly performed with the same loading arrangement in June 2016, March 2017 and March 2018.

## 3. Results and Discussion

This chapter describes and analyzes the results obtained from laboratory testing of concrete specimen and field measurements of the bridge structure.

### 3.1. Concrete Specimen Tested in Laboratory Conditions

**Figure 7:**
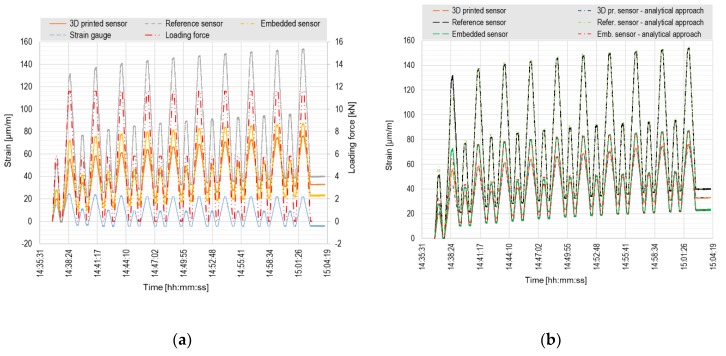
Cyclic loading test of concrete specimen: (**a**) Comparison of values measured by FBG sensors and strain gauge; (**b**) Comparison of measured and calculated values.

The plot in Figure 7a shows the course of loading force curve for cycling loading test with the response of both tested and reference sensors. The test consisted of 10 cycles with the same loading scheme. The beam was assumed to be loaded in the elastic part of the working diagram.

Due to the inaccurately estimated value of the maximum beam load-bearing capacity, the selected load steps were partly outside the elastic behavior. The main problem has been identified after the loading test—the concrete sample didn’t collapse in pure bending; it was a combination of shear-bending failure with the predominance of shear. The combination of the dimensions of the element, its material properties, and four-point test configuration (division of the element to the equal thirds) was unfavorably chosen. Shear load-bearing capacity was in this particular case lower than the load-bearing capacity in bending. Because of this, the first cracks were formed in the beam during the cyclic loading of the first load test.

**Figure 8:**
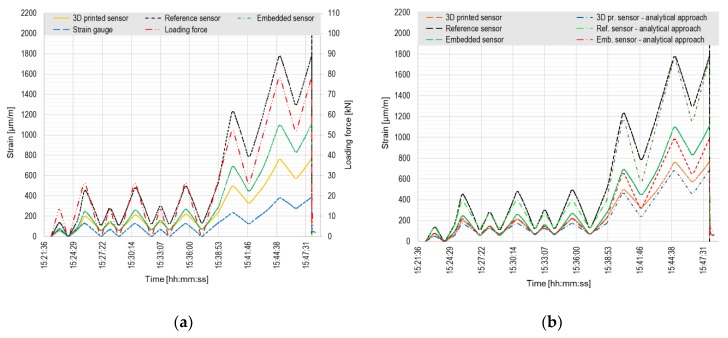
Destructive loading test of concrete specimen: (**a**) Comparison of values measured by FBG sensors and strain gauge; (**b**) Comparison of measured and calculated values.

Therefore, both tested and reference sensors showed a gradually increasing strain in each cycle. The course of all curves is almost identical, the absolute measured values, however, differ significantly. The explanation of the different measured values is quite simple; the position of sensors in the cross section regarding the neutral axis is not the same and is schematically presented in Figure 3. The reference sensor HBM was located closest to the bottom beam surface, the values of the measured strain in this sensor are therefore the biggest of all the sensors. The values measured by both tested sensors are very similar, differs by about 20%, the position of those sensors in the cross section differed by 15 mm (the distance from the neutral axis to the bottom fibers was 100 mm, i.e., 15 mm corresponds to 15%). The measured values of both the tested sensors are also significantly higher than the values indicated by the strain gauge. This may indicate a crack directly under the strain gauge or a mistake in a bonding process (the strain gauge was not perfectly coherent with the concrete surface). Therefore, the results of the strain gauge measurements were not included in the comparison of measured and calculated data (in Figure 7b and Figure 8b). The values measured by both tested sensors at maximum load differ by approximately 5%, the difference between the tested 3D printed sensor and the reference sensor is 8% and between the embedded FBG sensor and the reference sensor is 5%. After unloading, the offset of all sensors is observed. The stability of the sensors was probably influenced by the micro-cracks created by the plastic behavior of the beam during loading.

Figure 7b shows a comparison of the measured and analytically calculated values for the tested sensors and the reference HBM sensor. In order to be well comparable, the analytical calculations had to consider the unexpected plastic deformation of the beam (after unloading, the strain does not reach the zero value). The full lines show the data measured directly by FBG sensors. Considering the offset from creep and cracks in concrete, the analytically calculated data are displayed with a dot-and-dash lines. Therefore, it can be said that the data determined by the calculation considering cracks and creep are almost identical to the measured data for the relevant sensors.

A comparison of the measured and calculated values for the destructive loading test is presented in Figure 8.

Figure 8a presents that the highest strain values are indicated by the reference HBM sensor, as during the previous test. The position of the sensor regarding the neutral axis is significantly different than in case of the tested sensors—the distance from the sensor to the neutral axis is the highest. Both tested sensors give a similar trend of the measured values, which differ by about 30% at the time of the failure. The strain gauge gives the lowest values of the measured data, as in the previous test. When firstly loaded to ca. 16 kN, a major plastic deformation occurred in the sample. The second major cracking of the sample happened when loaded to ca. 30 kN.

Considering the analytical calculation, the elastic model of concrete behavior was employed in the first loading phase (the first three load cycles) with taking the permanent deformation into consideration. In the second phase of loading (gradually increasing loading force), a behavior model of concrete after cracks formation heading to the failure was considered. As can be seen from Figure 8b, the FBG sensors have quite good match with the analytical procedure. Considering the loading branch in the elastic part the values differ by 17% on average, the loading branch in the plastic part differs by 7% on average. The differences in the unloading branch are significantly higher due to inaccurate estimation of the permanent plastic deformation of the sample.

### 3.2. Testing of FBG Sensors in Concrete Bridge Structure

**Figure 9:**
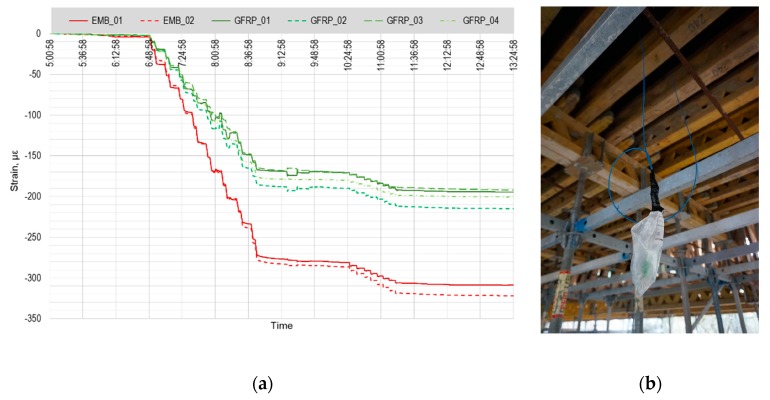
(**a**) Record of measured strains during the prestressing of the bridge. Shades of red – embedded FBG sensors, shades of green – four measuring points of GFRP sensor array. (**b**) Optical cable freely hanging through the wooden formwork.

All the sensors were installed into the bridge structure at the end of October 2017, the bridge has been concreted immediately after the installation and the monitoring of prestressing of the structure was provided on October 31, 2017. The plot in Figure 9a shows the main phase of prestressing as well as the phase of additional prestressing after stabilization. The embedded FBG sensors show generally bigger values than the GFRP sensing points; their position in relation to the neutral axis is different and that explains the offset (the embedded FBG sensors are placed closer to the bottom part of the cross section, the GFRP sensor array is closer to the neutral axis). All the sensing points registered well both prestressing phases as well as all the individual prestressing steps.

After the prestressing process, the monitoring system was disconnected and temporarily removed in order to enable the completion of all the construction works as smooth as possible. Six optical cables with sealed FC/APC connectors (two for each embedded FBG sensor and two for GFRP sensor array) were left freely hanging from the concrete structure (Figure 9b). The formwork was removed and heavy machinery came to provide the earthworks. During these works, five out of six connectors were damaged. Due to the combination of unfavorable weather conditions (heavy freezing and snowing) and the fact that optical fiber splicing is not possible at temperatures below 0 °C, the measurement system was repaired and completely restored in May 2018.

**Figure 10:**
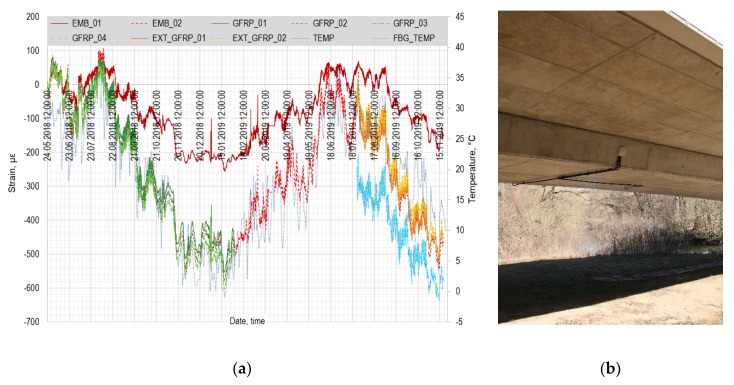
(**a**) Record of 18 months of bridge structure monitoring. Shades of red—embedded FBG sensors, shades of green—four measuring points of GFRP sensor array. Shades of yellow—external GRFP array. Shades of blue—recorded temperatures. (**b**) Small optical switchboard and optical cables in protective tubes mounted on the bridge structure.

On May 24, 2018 the long-term monitoring of the bridge structure started. All the necessary connectors were repaired and all the sensors reconnected. All the optical cables from the sensors are routed into the small optical switchboard, Figure 10b. A single underground armored optical cable connects the switchboard with the optical interrogator FBGuard 1550 placed inside a vacation property located a few hundred meters from the bridge structure. In the plot in Figure 9a, the embedded FBG sensors are presented in the shades of red and the measuring points in the GFRP array in the shades of green. The average day temperature has been obtained from the data of Czech Hydrometeorological Institute (dashed blue line). The data measured by the embedded FBG sensor 02 and by the whole GFRP array corresponds very well to the temperature changes. The embedded FBG sensor 01 gives the values in a good trend, but with significantly lower magnitudes—this is caused by the manufacturing defect.

The signal from the GFRP array was completely lost on February 06, 2019. The measuring line was checked by the OTDR tester that identified a defect on the optical fiber inside the bridge structure. Unfortunately, it is not possible to repair such a defect for obvious reasons. The most probable cause of the defect is that the array had been already slightly damaged during the concreting process of the bridge and eventually, in February, after a significant period of frosty nights, the fiber broke. The lesson learned from this damage is that 1 mm thick GFRP coating is not suitable for real-scale concrete structures. Indeed, the 3 mm thickness would be much more durable. Therefore, on July 24, 2019, the external GFRP array with two measuring points was attached to the bridge surface by the chemical anchor together with the FBG temperature sensor. The location of the measuring points approximately corresponds to the location of the original GFRP array (the measuring points lie in the same cross-sections; the distance from the neutral axis is obviously different). In Figure 10a, the external GFRP array is presented in the shades of yellow and the FBG temperature sensor by a bright blue dashed line. Regarding the measured strain, the external GFRP sensor corresponds very well with the embedded FBG sensor 02 and they both correspond well with the temperature changes. 

**Figure 11:**
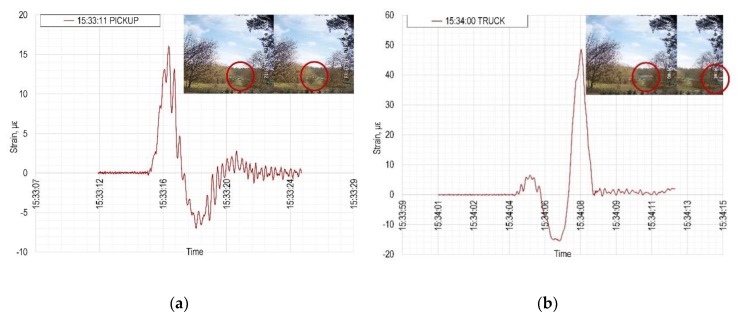
The automatic record of the response of the FBG sensor to (**a**) a pickup, (**b**) a truck crossing the instrumented bridge.

The presented monitoring system is suitable not only for long-term observation of impacts of temperature changes and possible deterioration of the load-bearing bridge structure, but may be very easily used for the monitoring of the traffic intensity. Figure 11 shows the response of one selected sensor to the passage of different kinds of vehicles in different directions. In Figure 11a, there was a pickup moving to the left; the sensor detected one big peak in magnitude around 15 με followed by a smaller peak of approximately 2 με. On the other hand, Figure 11b presents a passage of a truck moving to the right; first smaller peak reaching 6 με is in this case followed by a higher peak of magnitude around 48 με. For testing purposes, there is a low-resolution IP camera pointing towards the bridge with the automatic motion detection (photos in Figure 11). These photos serve as a temporary control system for the automatic record of the response of sensors to passing vehicles. 

### 3.3. Four-Point Bending Tests of Timber Beam

**Figure 12:**
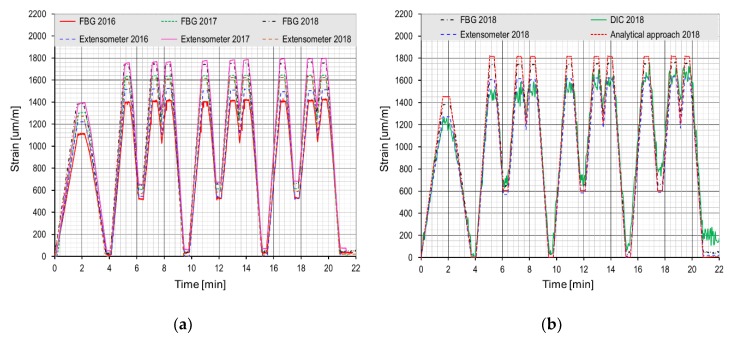
Cyclic loading tests of timber specimen: (**a**) Year-on-year comparison of data from the tests in years 2016, 2017 and 2018, [29]; (**b**) Comparison of measured and calculated values from test carried out in year 2018.

The FBG sensor and the extensometer placed in the middle of the timber beam span in the tensile zone of the cross section were chosen for the representative data comparison together with the DIC method and the analytical calculation method. In Figure 12, the measured and calculated values of strains are plotted as functions of time. From the year-to-year comparison of the data presented in Figure 12a, it can be seen that the strain of the beam increases every year at the same loading force. The increase of strain corresponds well to the decrease of the wood modulus of elasticity during the loading tests (13,636 MPa in 2016, 11,654 MPa in 2017, and 11,059 MPa in 2018). The decrease of the modulus of elasticity was caused by the gradually increasing plastic deformation of the wood due to the mechanical stress. From the measured data, an offset after full unloading of the test beam, when the stain is expected to be equal to zero is also evident. This offset grew slightly and at the end of each test it was about 40 μm/m. The year-on-year increase of the strain with the increasing offset during the tests was confirmed also by the reference extensometer sensors. The digital image correlation method, which is compared to the measured and calculated values in Figure 12b, was used as the second reference measurement for the test only in 2018. The laboratory testing was briefly presented in [29].

## 4. Conclusions

The paper presents laboratory comparative tests of concrete beam integrated FBG sensors together with results from sensor system deployment in the concrete bridge structure. The test concrete beam with integrated FBG sensors of different kinds and reference sensors was exposed to two standardized four-point bending tests. The test results show good coincidence between the sensors outputs during the load tests. Comparing the values measured by FBG sensors at maximum load it can be stated, that the difference between the two tested sensors is 5%, between the tested 3D printed sensor and the reference sensor is 8% and between the embedded FBG sensor and the reference sensor is 5%. The data measured by FBG sensors also coincide quite well with the data obtained by the analytical procedure. The values differ by 17% in average in the elastic part of the working diagram of the sample during the loading branch and by 7% in average in the plastic part of the working diagram. The differences in the unloading branch are significantly higher due to inaccurate estimation of the permanent plastic deformation of the sample. The test proved the applicability of FBG sensors for strain/load measurement integrated in the beam. The paper introduces the integration of FBG sensors into the structure of real-scale glued laminated timber within the manufacturing process. Due to the fact that the time period between the GLT beam manufacturing and its last experimental testing is two years, and the integrated fibers do not show any loss of sensitivity, it can be stated that there is no degradation of the fiber in time regarding the adhesive. Although the timber beam exhibits permanent plastic deformation, the embedded optical fibers adapted well with no slips in the glued layers. During the experimental loading tests, extensometers were used as the reference measurement for integrated FBGs. The highest measured relative error was 14%, with the exception of unloading the specimen to zero. The highest measured absolute error (160 μm/m) was achieved when loaded to 50% of the maximum load bearing capacity. It is expected that with higher load force the absolute error will increase and the relative error will decrease. This paper shows the results of real deployment of FBG measuring systems in a concrete bridge structure where embedded and GFRP FBG sensors were used. The deployment shows problems during installation phase where GFRP sensors were most likely partially damaged and stopped operation after eight months. The application of the sensors to the bridge structure shows the possibility to determine the overall load of the concrete structure, as well as the observation of traffic intensity, and a rough estimate of weights of passing vehicles. While FBG sensor technology is still quite expensive, GFRP coated sensors represent a new possibility for simple installation and monitoring of the structure from multiple points without the necessity to connect separate sensors to the measuring chain. With the decreasing price of FBG interrogation units, this technology is becoming available for deployment. The main advantage of the FBG integration is the protection from the ambient environment due to the integration into the monitored structure, but the robustness of the system is still an issue, specifically in the installation phase.

## References

[1] Lai M., Karalekas D., Botsis J. (2013). On the Effects of the Lateral Strains on the Fiber Bragg Grating Response. Sensors.

[2] Růžička M., Dvořák M., Doubrava K. Strain measurement with the Fiber Bragg Grating optical sensors. Proceedings of the 50th Annual Conference on Experimental Stress Analysis.

[3] Othonos A.S., Kyriacos K. (1999). Fiber Bragg gratings: Fundamentals and applications in telecommunications and sensing.

[4] Li D., Ren L., Li H., Song G. (2012). Structural Health Monitoring of a Tall Building during Construction with Fiber Bragg Grating Sensors. Int. J. Distrib. Sens. Netw..

[5] Biswas P., Bandyopadhyay S., Kesavan K., Parivallal S., Sundaram B., Ravisankar K., Dasgupta K. (2010). Investigation on packages of fiber Bragg grating for use as embeddable strain sensor in concrete structure. Sens. Actuators A Phys..

[6] Fang L., Chen T., Li R., Liu S. (2016). Application of Embedded Fiber Bragg Grating (FBG) Sensors in Monitoring Health to 3D Printing Structures. IEEE Sens. J..

[7] Hideaki I., Hiroshi Y., Akira M. (2001). Health monitoring system using FBG-based sensors for a 12-story building with column dampers. Proc. SPIE.

[8] Wacker J., Deza U., Phares B.M., Wipf T.J. Development of a smart timber bridge girder with fiber optic sensors. Proceedings of the International Conference on Timber Bridges.

[9] Li N., Jiang S., Wu M., Shen S., Zhang Y. (2018). Deformation Monitoring for Chinese Traditional Timber Buildings Using Fiber Bragg Grating Sensors. Sensors.

[10] Marsili R., Rossi G., Speranzini E. (2018). Fibre Bragg Gratings for the Monitoring of Wooden Structures. Materials.

[11] Xavier J., Fernandes J.R.A., Frazão O., Morais J.J.L. (2015). Measuring mode I cohesive law of wood bonded joints based on digital image correlation and fibre Bragg grating sensors. Compos. Struct..

[12] Tamaki K., Yuasa K., Morikawa H., Takemoto O. Verification of bridge monitoring system using FBG optical fiber sensors on existing prestressed concrete bridge. Proceedings of the fib Symposium: Concrete—Innovations in Materials, Design and Structures.

[13] Nishio M. (2017). Quality Evaluation of Fiber-Optic Strain Data Acquired in Long-Term Bridge Monitoring. Sens. Mater..

[14] Kim T.M., Kim D., Kim M.K., Lim Y.M. (2016). Fiber Bragg grating-based long-gauge fiber optic sensor for monitoring of a 60 m full-scale prestressed concrete girder during lifting and loading. Sens. Actuators A Phys..

[15] Adewuyi A.P., Wu Z., Franklin S.O. (2017). Performance monitoring of an existing concrete bridge using strain measurement data. Struct. Health Monit..

[16] Gebremichael Y.M., Li W., Meggitt B.T., Boyle W.J.O., Grattan K.T.V., McKinley B., Boswell L.F., Aarnes K.A., Aasen S.E., Tynes B. (2005). A Field Deployable, Multiplexed Bragg Grating Sensor System Used in an Extensive Highway Bridge Monitoring Evaluation Tests. IEEE Sens. J..

[17] Bonopera M., Chang K., Chen C., Lee Z., Sung Y., Tullini N. (2019). Fiber Bragg Grating-Differential Settlement Measurement System for Bridge Displacement Monitoring: Case Study. J. Bridge Eng..

[18] Sigurdardottir D.H., Glisic B. (2015). On-site validation of fiber-optic methods for structural health monitoring: Streicker Bridge. J. Civ. Struct. Health Monit..

[19] Suopajarvi P., Pennala R., Heikkinen M., Karioja P., Lyöri V., Myllylä R., Nissila S., Kopola H., Suni H. (1998). Fiber optic sensors for traffic monitoring applications. Proc. SPIE.

[20] Roveri N., Carcaterra A., Sestieri A. (2015). Real-time monitoring of railway infrastructures using fibre Bragg grating sensors. Mech. Syst. Sig. Process..

[21] Filograno M., Corredera P., Rodriguez-Barrios A., Martin-Lopez S., Rodriguez-Plaza M., Andres-Alguacil A., Gonzalez-Herraez M. (2012). Real-Time Monitoring of Railway Traffic Using Fiber Bragg Grating Sensors. IEEE Sens. J..

[22] Schulz W.L., Seim J.M., Udd E., Morrell M., Laylor H.M., McGill G.E., Edgar R. (1999). Traffic monitoring/control and road condition monitoring using fiber optic-based systems. Proc SPIE.

[23] Čápová K., Velebil L., Včelák J., Demuth J. Field Testing of FBG Based Deformation Sensors Embedded in Concrete Bridge Structure. Proceedings of the Conference CETRA 2018.

[24] Velebil L., Zelený R., Včelák J., Dvořák M., Kuklík P., Terebesyová M., Olbrich M. Optical Fibre Sensors as a Potential Solution for Monitoring Wooden Structures. Proceedings of the Conference WCTE 2016.

[25] Velebil L., Čápová K., Včelák J., Kuklík P., Demuth J., Dvořák M. Mechanical Stress Monitoring of Timber and Concrete Structures by Fibre Optic Sensors. Proceedings of the Conference WCTE 2018.

[26] FBGuard—Advanced Monitoring System. http://www.safibra.cz/en/fbguard-interrogation-unit.

[27] Záleský M., Záleský J., Šašek L., Čápová K. In-Situ Testing of FBG Deformation Sensors with Use of a New Test Beam. Proceedings of the 5th International Young Geotechnical Engineers’ Conference.

[28] European Committee for Standardization Timber Structures—Structural Timber and Glued Laminated Timber—Determination of Some Physical and Mechanical Properties. https://csnonline.agentura-cas.cz/Detailnormy.aspx?k=91855.

[29] Čápová K., Velebil L., Včelák J., Dvořák M., Šašek L. (2019). Environmental Testing of a FBG Sensor System for Structural Health Monitoring of Building and Transport Structures. Procedia Struct. Integrity.

